# Screening Mammography and Artificial Intelligence: A Comprehensive Systematic Review

**DOI:** 10.7759/cureus.79353

**Published:** 2025-02-20

**Authors:** Enas Abu Abeelh, Zain Abuabeileh

**Affiliations:** 1 Radiology, Primary Health Care Corporation, Doha, QAT; 2 Radiology, King Hussein Cancer Center, Amman, JOR

**Keywords:** ai-assisted diagnosis, artificial intelligence, breast cancer, cancer detection, screening mammography

## Abstract

Screening mammography is vital for early breast cancer detection, improving outcomes by identifying malignancies at treatable stages. Artificial intelligence has emerged as a tool to enhance diagnostic accuracy and reduce radiologists' workload in screening programs, though its full integration into clinical practice remains limited, necessitating a comprehensive review of its performance. This systematic review assesses artificial intelligence's effectiveness in screening mammography, focusing on diagnostic performance, reduction of false positives, and support for radiologists in clinical decision-making. A systematic search was conducted across PubMed, Embase, Web of Science, Cochrane Central, and Scopus for studies published between 2013 and 2024, including those evaluating artificial intelligence in mammography screening and reporting outcomes related to cancer detection, sensitivity, specificity, and workflow optimization. A total of 13 studies were analyzed, with data extracted on study characteristics, population demographics, artificial intelligence algorithms, and key outcomes. Artificial intelligence-assisted readings in screening mammography were found to be comparable or superior to traditional double readings by radiologists, reducing unnecessary recalls, improving specificity, and in some cases increasing cancer detection rates. Its integration into workflows showed potential for reducing radiologist workload while maintaining high diagnostic performance; however, challenges such as high false-positive rates and variations in artificial intelligence performance across patient subgroups remain concerns. Overall, artificial intelligence has the potential to enhance the efficiency and accuracy of breast cancer screening programs, and while it can reduce unnecessary recalls and alleviate radiologists' workloads, issues with false positives and demographic variations in accuracy highlight the need for further research. With ongoing refinement, artificial intelligence could become a valuable tool in routine mammography screening, augmenting radiologists' capabilities and improving patient care.

## Introduction and background

Screening mammography has been a critical tool in the early detection of breast cancer, significantly improving outcomes by identifying malignancies at a stage where treatment is more effective [1]. However, the integration of artificial intelligence (AI) into mammography screening has attracted increasing attention due to the potential to enhance diagnostic accuracy and reduce the workload of radiologists [2]. For instance, key concepts such as false positives (instances where benign findings are incorrectly flagged as suspicious), specificity (the ability of a test to correctly identify non-cancerous cases), and sensitivity (the ability to correctly detect cancer) are essential for understanding the performance of AI models in this context. In this review, "AI models" refer to computer algorithms, often based on deep learning techniques, that are trained to analyze mammography images and detect patterns associated with cancer. AI’s capacity to analyze vast amounts of image data quickly and accurately holds promise for improving the efficiency and effectiveness of breast cancer screening programs worldwide [3].

Despite its potential, the clinical adoption of AI in screening mammography has faced challenges. Studies have shown that AI can match or even surpass radiologists in specific diagnostic tasks, but its standalone use still presents limitations, particularly in complex cases [4]. These challenges-such as high false positive rates and workflow inefficiencies-underscore the need for a more thorough evaluation of AI’s real-world performance. A comprehensive evaluation of AI’s performance in real-world settings, including its sensitivity, specificity, and accuracy, is essential to fully understand its role in enhancing mammography screening [5].

Current literature demonstrates a gap in understanding AI's ability to reduce false positives and unnecessary recalls, as well as its effectiveness in distinguishing between benign and malignant findings. Studies highlight AI’s potential to reduce radiologist workload by identifying normal mammograms, but more prospective trials are required to validate these findings in diverse screening populations [6,7]. This review aims to address this gap by analyzing recent developments and identifying key areas where AI can be integrated into clinical practice to enhance screening efficiency and accuracy.

The objective of this systematic review is to comprehensively evaluate the application of AI in screening mammography, focusing on its ability to improve diagnostic performance, reduce false positives, and support radiologists in clinical decision-making. To achieve this, we aim to analyze various AI algorithms’ sensitivity, specificity, and overall effectiveness in comparison to traditional radiologist assessments [8]. The methodology will include an exhaustive review of published studies in this domain, utilizing both retrospective and prospective data from screening programs across different populations [9].

## Review

Methods

Search Strategy

A comprehensive systematic search was conducted to identify studies on AI applications in mammography screening published between January 2013 and December 2024. The databases used for this search included PubMed, Embase, Web of Science, Cochrane Central, and Scopus. The search was restricted to peer-reviewed studies published in English between January 2013 and December 2024. The keywords used for the search were a combination of terms related to both "artificial intelligence", "mammography", "breast cancer screening", and "machine learning". Boolean operators (AND, OR) were applied to ensure a comprehensive search.

Eligibility Criteria

Studies were included in the review if they were published between January 2013 and December 2024, written in English, focused on the use of artificial intelligence in mammography screening, contained original research (excluding reviews, meta-analyses, or editorials), and included outcomes relevant to AI-based performance in cancer detection (e.g., sensitivity, specificity, accuracy) or workflow optimization. Studies were excluded if they focused on AI applications unrelated to mammography screening, were published in a language other than English, did not present original research (i.e., opinion papers or commentaries), or were published before 2013.

Study Selection Process

The study selection process followed the PRISMA (Preferred Reporting Items for Systematic Reviews and Meta-Analyses) guidelines. After an initial database search, duplicates were removed. Titles and abstracts were screened for relevance by two independent reviewers. Full-text articles of potentially relevant studies were then retrieved and assessed for eligibility. Any disagreements during the selection process were resolved through discussion or consultation with a third reviewer.

The search initially was carried out using five databases: PubMed (n = 400), Embase (n = 250), Web of Science (n = 210), Cochrane Central (n = 83), and Scopus (n = 300), yielding a total of 1,243 records. After removing 353 duplicates, 890 articles were screened for relevance based on their titles and abstracts. Of these, 75 full-text articles were assessed for eligibility. A total of 62 articles were excluded for reasons such as insufficient reporting of key outcome measures (n = 39), methodological limitations that preclude robust data extraction (n = 15), and lack of relevant data (n = 8). Finally, 13 studies were included in this systematic review.

Data Extraction

Data extraction was performed independently by two reviewers using a standardized extraction form, and the extracted data included study characteristics (e.g., author, year, country), study design (e.g., cohort study, case-control study) where relevant, screening outcomes (e.g., cancer detection rates, recall rates, false-positive rates, specificity, sensitivity), and key findings related to artificial intelligence's performance and its role in improving mammography screening.

Data Synthesis

A narrative synthesis of the results from the included studies was performed. The focus was on qualitative analysis of AI's accuracy, sensitivity, specificity, false-positive rates, and its role in improving screening efficiency. Quantitative analysis was not performed, and no pooled sensitivity or specificity rates were calculated. Instead, individual studies' outcomes were compared and discussed based on their reported findings. Studies varied in methodology, and findings were summarized in tables where appropriate.

PRISMA Flow Diagram

**Figure 1:**
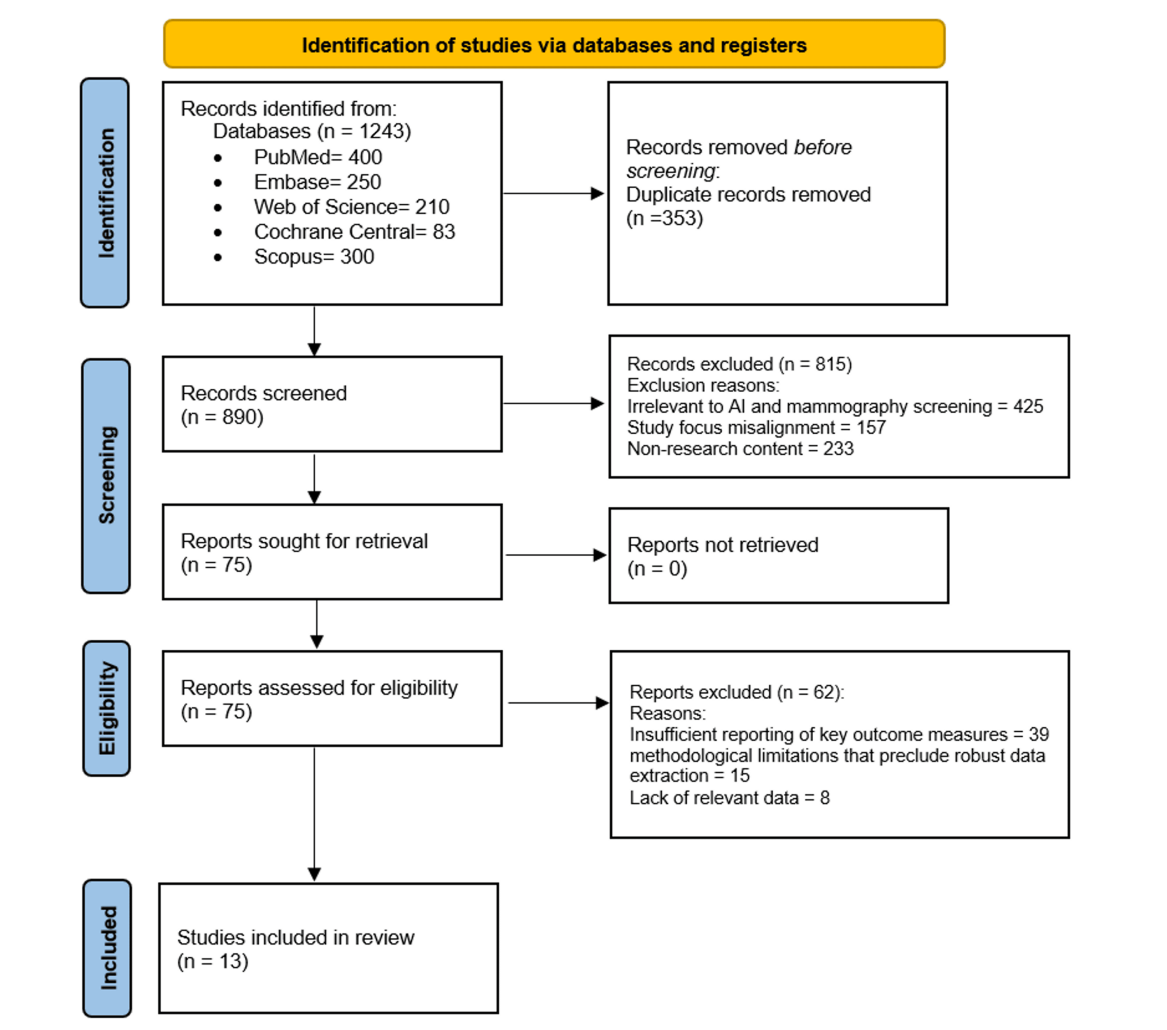
PRISMA Flow Diagram of Study Selection

The selection process for studies included in the systematic review is outlined in the PRISMA flow diagram as shown in Figure 1.

Results

The systematic review included 13 studies that comprehensively evaluated the application of AI in screening mammography. These studies assessed AI's performance in different aspects of breast cancer detection, diagnosis, workflow efficiency, and its integration into clinical practice. The included studies varied in methodology, ranging from prospective clinical trials to simulation studies and retrospective analyses, each providing valuable insights into AI's role in screening mammography (Table 1).

**Table 1:** Summary of Included Studies on AI in Screening Mammography CAD: computer-aided detection

Study Title	Author(s)	Journal Title	Place of Publication	Key Findings	Year
Artificial intelligence for breast cancer detection in screening mammography in Sweden: a prospective, population-based study	Dembrower et al. [10]	The Lancet Digital Health	England	AI-assisted screening detected 4% more cancers compared to double reading by two radiologists. AI-based single reading was comparable to double reading by two radiologists, demonstrating AI's potential to replace a radiologist in the screening process.	2023
Artificial Intelligence (AI) for Screening Mammography, From the AJR Special Series on AI Applications	Lamb et al. [11]	American Journal of Roentgenology	United States	The study reviewed commercial AI algorithms for screening mammography, discussing their clinical applications and potential ethical considerations. The study highlighted the need for further clinical validation of AI algorithms in screening settings.	2022
Artificial Intelligence in Screening Mammography: A Population Survey of Women's Preferences	Ongena et al. [12]	Journal of the American College of Radiology	United States	A survey revealed that 77.8% of women supported the involvement of radiologists in AI-assisted screenings. However, only a small proportion supported the complete replacement of radiologists by AI, demonstrating the public's preference for human involvement in decision-making.	2021
Radiologist Preferences for AI-Based Decision Support During Screening Mammography Interpretation	Hendrix et al. [13]	Journal of the American College of Radiology	United States	Most radiologists expressed interest in using AI if its sensitivity and specificity were balanced. However, radiologists emphasized the importance of using AI tools that complement their work, rather than fully replacing radiologists, to maintain diagnostic accuracy.	2022
Use of Artificial Intelligence for Reducing Unnecessary Recalls at Screening Mammography	Kim et al. [14]	Korean Journal of Radiology	Korea (South)	AI-aided screening reduced unnecessary recall rates and improved specificity while maintaining high sensitivity for cancer detection. The study demonstrated AI's effectiveness in reducing radiologists' workload by lowering the number of false-positive cases.	2022
Diagnostic performance with and without AI assistance in real-world screening mammography	Lee et al. [15]	European Journal of Radiology Open	England	The study found no significant difference in cancer detection rates between radiologists with and without AI assistance. However, the AI-CAD system improved specificity and accuracy while reducing recall rates, demonstrating its potential to optimize screening workflows.	2024
AI-Based CAD in Mammographic Interpretation Workflow	Yoon et al. [4]	European Journal of Radiology Open	England	AI-CAD detected 17.9% additional cancers that were initially missed by radiologists. However, it increased recall rates and flagged 89.0% of marks as false positives, indicating the need for further improvement in AI specificity to avoid unnecessary recalls.	2023
Frequency and Characteristics of Errors by AI in Reading Screening Mammography	Zeng et al. [16]	Breast Cancer Research and Treatment	Netherlands	The study systematically reviewed AI errors in screening mammography. False-positive rates decreased with increasing positivity thresholds, while false negatives increased. Reporting on other error types (e.g., location errors) was sparse, highlighting a gap in current AI evaluations.	2024
Effect of Benign Biopsy Findings on AI-Based Cancer Detection in Screening Mammography	Zouzos et al. [17]	JMIR Publications	Canada	The study found that AI systems flagged a higher proportion of women with previous benign biopsy findings, indicating that prior biopsy data should be considered in AI model training to prevent unnecessary recalls in future screenings.	2023
External Validation of AI Algorithms for Automated Interpretation of Screening Mammography	Anderson et al. [18]	Journal of the American College of Radiology	United States	Independent validation studies showed that AI algorithms generally improved accuracy compared to radiologists alone. However, the studies revealed concerns regarding potential bias in patient selection and the quality of the reference standards used in AI algorithm evaluations.	2022
Comparative Performance of AI Algorithms for Screening Mammography	Taya [19]	Radiology. Imaging Cancer	United States	The study compared several AI algorithms for breast cancer detection and found that combined human and AI interpretation was superior to AI-alone, particularly in cases of high-grade tumors where AI exhibited higher sensitivity.	2020
Use of Novel AI-Based CAD for Screening Mammography	Heywang-Kobrunner et al. [20]	Acta Radiologica	England	The AI system achieved similar cancer detection rates to human readers but had lower specificity. Combining human and AI interpretations increased sensitivity, but required consensus readings for more cases, reducing the time saved by AI automation.	2023
AI for Interval Breast Cancer Detection at Screening Mammography	Nanaa et al. [21]	Radiology	United States	The AI system showed improved cancer detection in cases missed by human readers, especially in node-positive cancers. However, its accuracy in localizing the lesions was limited, highlighting the need for further refinement of AI systems to improve lesion localization accuracy.	2024

Narrative Results

The systematic review indicates that AI may enhance the diagnostic performance of screening mammography. Several studies, such as Dembrower et al. (2023) and Kim et al. (2022), reported that AI-assisted readings were associated with reduced false positives, lower recall rates, and improved specificity, which could potentially decrease the workload for radiologists [10,14]. In particular, the study by Kim et al. (2022) observed that AI use in screening mammography was linked to a reduction in unnecessary recalls without compromising cancer detection rates, suggesting a possible role for AI in increasing the efficiency and accuracy of mammography screening [14].

Risk of Bias Assessment

Table 2 provides an overview of each study’s type, risk of bias, and relevant methodological notes to enhance transparency.

**Table 2:** Risk of Bias Assessment for Included Studies CAD: computer-aided detection

Reference	Study	Study Type	Overall Risk of Bias	Notes/Rationale
Dembrower et al. (2023) [10]	Prospective, population-based study	Prospective study	Low	Well-designed; robust methodology with clear outcome measures and minimal confounding.
Lamb et al. (2022) [11]	Review of commercial AI algorithms	Narrative review	Moderate	Comprehensive review; however, lacks a formal bias assessment and detailed protocol registration.
Ongena et al. (2021) [12]	Population survey of women's preferences	Survey study	Low	Clear methodology and sampling; potential self-report bias minimized by large sample size.
Hendrix et al. (2022) [13]	Radiologist preferences for AI-based decision support	Survey study	Low	Well-structured survey with representative sample; minor risk of selection bias.
Kim et al. (2022) [14]	Simulation study on reducing unnecessary recalls	Simulation/retrospective study	Moderate	Retrospective design with simulation limits generalizability; potential confounders partially addressed.
Lee et al. (2024) [15]	Diagnostic performance with and without AI assistance	Real-world screening study	Moderate	Real-world data with inherent retrospective limitations; some concerns regarding blinding and confounders.
Yoon et al. (2023) [4]	AI-Based CAD in mammographic interpretation workflow	Observational study	Moderate	Increased recall rates and high false positives; potential bias in patient selection noted.
Zeng et al. (2024) [16]	Review of AI errors in reading screening mammography	Systematic review	Moderate	Provides a systematic review; however, reporting on certain error types is limited.
Zouzos et al. (2023) [17]	Effect of benign biopsy findings on AI-based cancer detection	Retrospective case-control study	Low	Clear design with adequate control for confounders; retrospective nature noted but minimized risk overall.
Anderson et al. (2022) [18]	External validation of AI algorithms for automated interpretation	Validation study	Moderate	Addresses validation across independent datasets; concerns remain regarding patient selection bias.
Taya (2020) [19]	Comparative performance of AI algorithms	Comparative study	Low	Well-controlled comparison between AI and combined human-AI interpretation; minimal bias observed.
Heywang-Kobrunner et al. (2023) [20]	Use of novel AI-based CAD for screening mammography	Observational study	Moderate	Comparable detection rates but lower specificity; potential limitations in consensus reading reported.
Nanaa et al. (2024) [21]	AI for interval breast cancer detection	Observational study	Moderate	Improved detection in missed cases but limited by suboptimal lesion localization; moderate overall risk.

A structured risk of bias assessment was conducted for each included study using pre-defined criteria covering factors such as study design, patient selection, performance of the index test, use of the reference standard, and the timing of outcome measurements. Two independent reviewers evaluated these aspects for each study, and any disagreements were resolved through discussion; if consensus was not reached, a third reviewer was consulted for a final decision. Overall, many studies were rated as having a moderate risk of bias, primarily due to their retrospective design, potential selection bias, and limitations in methodological reporting.

However, AI systems are not without limitations. Zeng et al. (2024) reviewed the types of errors made by AI systems, emphasizing that false positives and false negatives were still significant concerns, particularly at lower positivity thresholds [16]. False positives, in particular, increased the number of unnecessary recalls, as evidenced by Yoon et al. (2023), who reported that 89% of AI-detected abnormalities were ultimately benign [4]. Despite these challenges, AI consistently demonstrated improved sensitivity for detecting breast cancer, with studies like Heywang-Kobrunner et al. (2023) showing that AI can achieve comparable detection rates to human double reading [20].

Importantly, public and radiologist acceptance of AI integration into clinical workflows remains mixed. Ongena et al. (2021) found that a significant portion of the general population still favored human oversight, while Hendrix et al. (2022) noted that radiologists preferred AI systems that assist rather than replace them, particularly those that complement their review process [12,13].

These findings underscore AI's promise in improving the efficiency and accuracy of breast cancer screening but also highlight the need for continued refinement in reducing error rates and addressing public and professional concerns about full AI implementation. Further prospective studies and external validation are required to optimize AI performance and integrate it into routine clinical practice.

Discussion

The results of this systematic review highlight the transformative potential of artificial intelligence (AI) in the context of screening mammography, showing promise in enhancing diagnostic accuracy, reducing unnecessary recalls, and supporting radiologists in clinical decision-making. While AI's integration into mammography workflows presents opportunities for improved screening efficiency, the findings also indicate a range of challenges, limitations, and areas for further research and refinement.

AI's Diagnostic Potential in Screening Mammography

The collective evidence from the reviewed studies underscores AI's potential to enhance the sensitivity and specificity of breast cancer detection in screening mammography. For instance, McKinney et al. (2020) conducted an extensive international evaluation of an AI system and reported that its sensitivity and specificity were comparable to, and in some settings even exceeded, those of experienced radiologists [3]. Similarly, Lauritzen et al. (2022) found that incorporating AI into the screening process maintained diagnostic accuracy while reducing false-positive rates [7]. These findings are further corroborated by more recent research, including the study by Dembrower et al. (2023) [10], which demonstrated that AI-assisted mammography screenings detected 4% more cancers compared to traditional double readings by radiologists. Collectively, these studies suggest that AI could serve as an effective adjunct in high-volume screening settings, potentially improving cancer detection rates and optimizing radiologist workload [3,7,10].

Despite these promising results, AI’s diagnostic potential must be considered within the broader context of radiologists' oversight. In the study by Ongena et al. (2021), many women expressed a preference for human radiologist involvement due to concerns about trust and communication; they felt that a human expert could better explain uncertainties and offer empathetic support during the screening process [12]. Similarly, radiologists in Hendrix et al. (2022) highlighted that a fully automated system might overlook the nuanced interpretation of imaging findings and individual patient histories, which are critical in complex cases [13]. These concerns contribute to the overall sentiment that, while AI may enhance detection capabilities, its use as a standalone tool is not yet widely supported, and human oversight remains essential to ensure diagnostic accuracy, accountability, and patient confidence.

AI and Reduction of Unnecessary Recalls

One of the most significant findings in this review is AI's potential to reduce unnecessary recalls, a critical concern in mammography screening that can lead to patient anxiety and increased healthcare costs. In the study by Kim et al. (2022), which involved 793 women recalled for supplemental mammographic views, the reader-averaged recall rate decreased significantly from 60.4% (95% CI, 57.8%-62.9%) to 49.5% (95% CI, 46.5%-52.4%) with AI aid (p < 0.001), while sensitivity for cancer detection remained comparable [14]. In addition, Zouzos et al. (2023) assessed the impact of prior benign biopsy findings on AI performance and found that the AI system flagged 3.5% of healthy women without a benign biopsy compared to 11% of healthy women with a benign biopsy [17]. Notably, for women with a benign biopsy, the AI flagging rate (8.5%) was similar to that of radiologists. These studies not only demonstrate a quantitative reduction in recalls with AI integration but also highlight how adjustments in the algorithm can address false positive concerns across different patient subgroups.

However, it is important to note that not all studies aligned with this trend. Yoon et al. (2023) reported that although AI improved cancer detection rates, it also increased the recall rate significantly [4]. In this study, 89.0% of the artificial intelligence-based computer-aided detection (AI-CAD) marks were observed on negative examinations. This high recall rate can be attributed to the algorithm's prioritization of sensitivity over specificity; the preset threshold (abnormality score ≥10%) led the system to flag subtle findings that radiologists might typically dismiss. Notably, 41.2% of the AI-CAD marks were retrospectively deemed negligible, highlighting that many of the additional recalls were due to false positives. These findings underscore the need for further refinement of AI algorithms to better balance sensitivity with specificity.

Discrepancies and Theoretical Implications

While the majority of studies point to AI’s benefits, a few discrepancies arise, particularly regarding its standalone performance. For instance, Lee et al. (2024) found that the diagnostic performance of radiologists did not significantly differ when AI assistance was provided, raising questions about AI’s actual value in real-world clinical settings [15]. Similarly, Nanaa et al. (2024) found that while AI could detect interval cancers missed by radiologists, its localization accuracy remained suboptimal, suggesting that AI may require further fine-tuning, particularly in lesion detection and characterization [21].

These discrepancies indicate that while AI holds substantial promises, its effectiveness may vary depending on factors such as the specific algorithm used, the dataset on which it is trained, and the experience of the radiologists using the tool. As noted by Braithwaite et al. (2024), the variability in AI’s performance across different settings underscores the need for standardized validation protocols and larger, multi-institutional studies to ensure consistent results [22].

Theoretically, the integration of AI into mammography screening could also lead to significant paradigm shifts in how screening programs are structured. For example, Lauritzen et al. (2022) propose that AI could enable more personalized screening strategies, where the frequency of mammograms is tailored to an individual's risk profile, potentially reducing over-screening and its associated harm [7].

Practical Applications and Future Directions

From a practical perspective, AI's role in reducing the workload of radiologists is one of its most promising applications. Several studies have reported that AI algorithms can effectively triage normal mammograms, thus decreasing the number of cases requiring detailed radiologist review. For example, Hickman et al. (2021) noted that, in triage applications, AI systems were able to correctly identify between 17% and 91% of normal mammograms, suggesting a substantial potential to reduce radiologist workload without significantly compromising cancer detection [2]. Similarly, Freeman et al. (2021) found that when AI was used as a screening tool, studies reported that 45% to 53% of women at low risk could be safely excluded from further radiologist review [6]. This reduction in workload not only streamlines the screening process but also allows radiologists to focus their expertise on more complex or ambiguous cases, thereby improving diagnostic efficiency and potentially alleviating burnout--a growing concern in the field.

Despite its promise, the full clinical adoption of AI in mammography screening is hindered by several practical, regulatory, and ethical concerns. For instance, recent large-scale studies, such as the nationwide PRAIM study in Germany by Eisemann et al. 2025, have demonstrated that AI-supported reading can increase cancer detection rates by 17.6% without compromising recall rates [23]. However, these promising results also underscore the regulatory hurdles that must be overcome. Regulatory approval requires rigorous evidence of safety and efficacy, along with continuous postmarketing surveillance to ensure sustained performance. Moreover, issues such as data privacy, the potential for algorithmic bias, and the lack of transparency in AI decision-making processes remain significant barriers, as noted by Retson and Eghtedari (2023) [24]. Additionally, as Zeng et al. (2024) pointed out, AI systems are not immune to errors, and accountability remains ambiguous-whether it lies with the AI developer, the healthcare provider, or the radiologist [16]. Together, these challenges highlight the need for comprehensive strategies that address both regulatory and operational aspects to fully integrate AI into clinical practice.

## Conclusions

In conclusion, the results of this systematic review suggest that AI has the potential to significantly enhance screening mammography by improving cancer detection rates, reducing unnecessary recalls, and supporting radiologists in their diagnostic decision-making. However, the current state of AI technology is not without limitations, particularly in terms of specificity and public acceptance of AI as an independent tool. While AI is unlikely to replace radiologists in the near future, its role as an adjunctive tool appears promising and could lead to substantial improvements in breast cancer screening programs. Future research should focus on refining AI algorithms to reduce false-positive rates, standardizing validation protocols across institutions, and addressing the ethical and practical challenges associated with AI adoption in clinical settings. Additionally, emerging evidence suggests that AI can markedly decrease the number of mammograms requiring radiologist review by accurately identifying normal cases, thereby enabling radiologists to concentrate on more complex cases and potentially reducing burnout.

## References

[1] Kim HE, Kim HH, Han BK (2020). Changes in cancer detection and false-positive recall in mammography using artificial intelligence: a retrospective, multireader study. Lancet Digit Health.

[2] Hickman SE, Woitek R, Le EP (2022). Machine learning for workflow applications in screening mammography: systematic review and meta-analysis. Radiology.

[3] McKinney SM, Sieniek M, Godbole V (2020). International evaluation of an AI system for breast cancer screening. Nature.

[4] Yoon JH, Han K, Suh HJ, Youk JH, Lee SE, Kim EK (2023). Artificial intelligence-based computer-assisted detection/diagnosis (AI-CAD) for screening mammography: Outcomes of AI-CAD in the mammographic interpretation workflow. Eur J Radiol Open.

[5] Tan XJ, Cheor WL, Lim LL, Ab Rahman KS, Bakrin IH (2022). Artificial intelligence (AI) in breast imaging: a scientometric umbrella review. Diagnostics (Basel).

[6] Freeman K, Geppert J, Stinton C, Todkill D, Johnson S, Clarke A, Taylor-Phillips S (2021). Use of artificial intelligence for image analysis in breast cancer screening programmes: systematic review of test accuracy. BMJ.

[7] Lauritzen AD, Rodríguez-Ruiz A, von Euler-Chelpin MC (2022). An artificial intelligence-based mammography screening protocol for breast cancer: Outcome and radiologist workload. Radiology.

[8] Strohm L, Hehakaya C, Ranschaert ER, Boon WP, Moors EH (2020). Implementation of artificial intelligence (AI) applications in radiology: hindering and facilitating factors. Eur Radiol.

[9] Yue W, Wang Z, Chen H, Payne A, Liu X (2018). Machine learning with applications in breast cancer diagnosis and prognosis. Designs.

[10] Dembrower K, Crippa A, Colón E, Eklund M, Strand F (2023). Artificial intelligence for breast cancer detection in screening mammography in Sweden: a prospective, population-based, paired-reader, non-inferiority study. Lancet Digit Health.

[11] Lamb LR, Lehman CD, Gastounioti A, Conant EF, Bahl M (2022). Artificial intelligence (AI) for screening mammography, from the AJR special series on AI applications. AJR Am J Roentgenol.

[12] Ongena YP, Yakar D, Haan M, Kwee TC (2021). Artificial intelligence in screening mammography: A population survey of women's preferences. J Am Coll Radiol.

[13] Hendrix N, Lowry KP, Elmore JG (2022). Radiologist preferences for artificial intelligence-based decision support during screening mammography interpretation. J Am Coll Radiol.

[14] Kim YS, Jang MJ, Lee SH (2022). Use of artificial intelligence for reducing unnecessary recalls at screening mammography: A simulation study. Korean J Radiol.

[15] Lee SE, Hong H, Kim EK (2024). Diagnostic performance with and without artificial intelligence assistance in real-world screening mammography. Eur J Radiol Open.

[16] Zeng A, Houssami N, Noguchi N, Nickel B, Marinovich ML (2024). Frequency and characteristics of errors by artificial intelligence (AI) in reading screening mammography: a systematic review. Breast Cancer Res Treat.

[17] Zouzos A, Milovanovic A, Dembrower K, Strand F (2023). Effect of Benign Biopsy Findings on an Artificial Intelligence-Based Cancer Detector in Screening Mammography: Retrospective Case-Control Study. JMIR AI.

[18] Anderson AW, Marinovich ML, Houssami N (2022). Independent external validation of artificial intelligence algorithms for automated interpretation of screening mammography: A systematic review. J Am Coll Radiol.

[19] Taya M (2020). Comparative performance of artificial intelligence algorithms for screening mammography. Radiol Imaging Cancer.

[20] Heywang-Köbrunner SH, Hacker A, Jänsch A (2023). Use of novel artificial intelligence computer-assisted detection (AI-CAD) for screening mammography: an analysis of 17,884 consecutive two-view full-field digital mammography screening exams. Acta Radiol.

[21] Nanaa M, Gupta VO, Hickman SE (2024). Accuracy of an artificial intelligence system for interval breast cancer detection at screening mammography. Radiology.

[22] Braithwaite D, Karanth SD, Divaker J (2024). Evaluating ChatGPT’s accuracy in providing screening mammography recommendations among older women: Artificial intelligence and cancer communication [PREPRINT]. Res Sq.

[23] Eisemann N, Bunk S, Mukama T (2025). Nationwide real-world implementation of AI for cancer detection in population-based mammography screening. Nat Med.

[24] Retson TA, Eghtedari M (2023). Expanding horizons: The realities of CAD, the promise of artificial intelligence, and machine learning’s role in breast imaging beyond screening mammography. Diagnostics (Basel).

